# ALG3 as a prognostic biomarker and mediator of PD-1 blockade resistance in hepatocellular carcinoma

**DOI:** 10.3389/fimmu.2025.1589153

**Published:** 2025-05-22

**Authors:** Pengju Tang, Zhenwei Han, Yiming Zhao, Tianxin Xu, Yu Zhang, Lirong Zhu, Fei Song, Cheng Gao, Jinbo Gong, Ji Cheng, Chenggui Wang, Tianlun Wang, Jie Xu, Yu Wang, Zhong Chen

**Affiliations:** ^1^ Department of Hepatobiliary Surgery, Affiliated Hospital of Nantong University, Medical School of Nantong University, Nantong, China; ^2^ Department of General Surgery, Jianhu County People’s Hospital, Yancheng, China

**Keywords:** hepatocellular carcinoma (HCC), ALG3(Asparagine-linked glycosylation 3), multiplex immunohistochemistry, immunotherapy, tumor microenvironment

## Abstract

**Background:**

Hepatocellular carcinoma (HCC) is a leading cause of cancer-related death globally, characterized by high heterogeneity and drug resistance, which significantly impacts clinical outcomes. The tumor microenvironment (TME) plays a critical role in HCC initiation and progression, with immune cell infiltration and immune checkpoint expression closely linked to tumor prognosis. N-glycosylation of proteins modulates immune responses within the TME. ALG3, a key N-glycosylation enzyme, is involved in protein glycosylation. Although ALG3 expression has been studied in various tumors, its role in regulating the immune microenvironment and its prognostic significance in HCC remain unclear.

**Methods:**

This study comprehensively evaluates ALG3 expression in HCC and its relationship with the immune microenvironment using various techniques. First, bioinformatics analysis of HCC-related data from the TCGA database was performed to investigate ALG3 expression patterns in tumor tissues and its correlation with clinical features. Multiplex immunohistochemistry (mIHC) was then used to validate ALG3 expression in HCC tissue samples and examine its relationship with immune cell infiltration. Additionally, cell experiments and 3D human organoid-based culture models were employed to further assess the role of ALG3 in the HCC immune microenvironment.

**Results:**

The results showed significant overexpression of ALG3 in HCC tissues, with high expression correlating significantly with poor tumor prognosis. Further analysis revealed that high ALG3 expression was associated with reduced infiltration of CD8^+^ T cells and CD68^+^ macrophages in both tumor and stromal areas, while positively correlating with increased infiltration of FOXP3^+^ regulatory T cells (Tregs). Notably, ALG3 expression levels were also positively correlated with PD-L1 expression in HCC tissues.

**Conclusions:**

ALG3 may serve as a potential prognostic biomarker and an immunotherapy target in HCC.

## 1 Introduction

Hepatocellular carcinoma (HCC) is one of the most common and lethal malignancies worldwide (1, 2). Treatment decisions for HCC are typically based on molecular subtypes and clinical staging. Traditional therapeutic approaches, such as surgical resection, liver transplantation, local ablation, and targeted therapies, have shown limited efficacy due to tumor heterogeneity and drug resistance, highlighting the urgent need for new strategies to improve long-term prognosis (3, 4).

Recent studies have revealed the complex role of the immune microenvironment in HCC initiation and progression. Immune cells, once thought to primarily inhibit tumor growth, are now recognized for their dual roles in both suppressing tumor growth and promoting immune evasion (5). This evolving understanding has led to the application of immunotherapeutic strategies, particularly immune checkpoint inhibitors (ICIs), which have shown promising clinical potential in patients with poor prognosis or those unresponsive to conventional therapies (6).

The tumor microenvironment (TME) plays a crucial role in the progression and prognosis of HCC. A comprehensive analysis of tumor-infiltrating immune cells (TIICs) provides valuable insights into immune evasion mechanisms and can guide the development of novel therapeutic approaches (7, 8). By integrating immune infiltration data with molecular and histological information, a more comprehensive understanding of HCC subtypes can be achieved, thus facilitating the advancement of personalized treatment strategies.

In recent years, the role of endoplasmic reticulum-associated glycosylation in the development of HCC has gained increasing attention. ALG3 (Asparagine-Linked Glycosylation 3) is a key enzyme involved in the N-glycosylation process that affects protein glycosylation and plays an important role in tumor cell survival and immune microenvironment regulation (9). ALG3 has been found to be abnormally expressed in several cancers, including bladder, ovarian, and breast cancers (9–11). Glycosylation modifications are closely linked to tumor resistance, immune evasion, and microenvironment remodeling (12–14). This study aims to investigate the role of ALG3 in the immune microenvironment of HCC and evaluate its potential as a prognostic and immunological biomarker.

## 2 Methods

### 2.1 Data collection and processing

The Cancer Genome Atlas (TCGA) database (https://portal.gdc.cancer.gov/) provides clinical and RNA sequencing data from pan-cancer patients. In this study, R software (with R packages such as Limma for differential expression analysis, survival and survminer for Kaplan-Meier survival analysis, and pROC for ROC curve analysis) was used to compare the ALG3 mRNA expression levels between adjacent and tumor tissues in hepatocellular carcinoma (HCC) patients from TCGA. Kaplan-Meier survival analysis was performed to assess the prognosis of 374 HCC patients from TCGA. Additionally, receiver operating characteristic (ROC) curves were plotted and the area under the curve (AUC) was calculated to evaluate the diagnostic value of ALG3 in HCC. Furthermore, external validation of ALG3’s prognostic value was conducted using two widely accepted online survival analysis platforms: LOGpc (https://bioinfo.henu.edu.cn/index.html) and KM-Plotter (https://kmplot.com/analysis/) (15, 16).

### 2.2 Clinical tissue samples

180 primary HCC patient samples were collected by the Department of Hepatic Surgery at the Affiliated Hospital of Nantong University between February 2022 and December 2024 for tissue microarray (TMA) analysis. The inclusion criteria were as follows: (I) no treatment prior to surgery and (II) postoperative pathological confirmation of primary HCC. All participants provided informed consent, and the study received approval from the Ethics Committee of the Affiliated Hospital of Nantong University (No. 2019-K021) (17).

### 2.3 Immune cells infiltration analysis

Based on TCGA data, the correlation between ALG3 mRNA expression and the abundance of tumor-infiltrating immune cells (TIICs) was further investigated using relevant modules from the OStme platform (https://bioinfo.henu.edu.cn/immune/immune.html) (18).

### 2.4 IHC

The pathological slides from 12 patients were first deparaffinized using xylene and then rehydrated through a series of alcohol solutions. For antigen retrieval, the slides were immersed in citrate buffer (10 mM, pH 6.0) and heated in a microwave. Afterward, the slides were treated with 3% hydrogen peroxide for 20 minutes to block endogenous peroxidase activity. To prevent nonspecific binding, the slides were incubated with 5% bovine serum albumin for 1 hour. An experienced pathologist, blinded to the clinical details of the patients, assessed the immunohistochemical (IHC) staining using a semi-quantitative H-score system. Staining intensity was categorized into four levels: 0 for no staining, 1 for weak staining, 2 for moderate staining, and 3 for strong staining. The positive rate was recorded on a scale from 0 to 100, and both staining intensity and percentage were evaluated, with the final score ranging from 0 to 300 (19).

### 2.5 mIHC

The tissue microarray (TMA) slides were initially deparaffinized with xylene and rehydrated in a series of alcohol and water solutions. Antigen retrieval was carried out by microwaving the slides in AR6 buffer. After blocking with a blocking buffer for 10 minutes, primary and secondary antibodies were applied sequentially. Following secondary antibody incubation, multiplex immunohistochemistry (mIHC) staining was performed with heat-induced antigen retrieval. To amplify the signals, protein stone fluorescence conjugated to an amide-linked fluorophore was used. Nuclei were stained with 4,6-diamidino-2-phenylindole (DAPI) (F6057, Sigma), and the slides were then sealed. The stained slides were scanned using the Vectra 3.0 automated quantitative pathology imaging system (PerkinElmer, USA) for further analysis. Tumor and stromal cores were captured using a ×20 Olympus objective lens. Using inForm^®^ cell analysis software (version 4.1.0, PerkinElmer), machine learning algorithms were employed to segment the images into cancerous and stromal cell regions, with single cells being segmented based on DAPI counterstaining. The pathologist set thresholds for each marker to ensure accuracy above 95% (20). Antibody details are provided in **Supplementary Table S1**.

### 2.6 Patient-derived organotypic tissue spheroids in a microfluidic chip-based 3D culture system

From January 2024 to March 2025, we obtained PDOTs from HCC specimens of 12 patients at the Affiliated Hospital of Nantong University. All patients in this cohort (n = 12) had not received chemotherapy, radiotherapy, or any anti-tumor treatment prior to specimen collection. All participants provided informed consent, allowing the use of their tissue samples and clinical data. The ethics approval number is: 2025-L102. Freshly excised postoperative tissue samples (approximately 1 cm³) were washed with PBS, minced, and transferred into a 15 mL centrifuge tube containing 5 mL of digestion solution, which consisted of Advanced DMEM/F-12 medium, 0.1% collagenase type IV, 0.05% hyaluronidase, and 0.01% deoxyribonuclease. The mixture was incubated at 37°C with agitation at 100 rpm. Every 15 minutes, the tissue digestion and formation of PDOTs were monitored.

After digestion, the PDOTs suspension was filtered through 40 μm and 100 μm cell strainers, retaining spheroids with diameters ranging from 40 to 100 μm. These spheroids were mixed with 10×PBS, 0.5M NaOH, and type I mouse tail collagen in a predetermined ratio and then pipetted into a microfluidic chip. After a 30-minute incubation at 37°C, drug treatments were applied: control group and anti-PD-1 (5 μM) treatment.

After 5–7 days of drug treatment, the culture medium was carefully removed, and the PDOTs were stained with 10 μL of AO/PI solution (Nexcelom Bioscience) under dark conditions at 4°C for 5 minutes. Fluorescence microscopy was used to distinguish live (green) and dead (red) cells. Cell viability was quantified using Image J software by analyzing the balance between red and green fluorescence intensities. The Tumor Killing Index (TKI) was calculated as follows:


$$\text{Alive}=\frac{AG}{AG+AR}$$

$$\text{ΔAlive}={\text{Alive}}_{NC}-{\text{Alive}}_{T}$$

$$TKI=\frac{\text{ΔAlive}}{\text{Alive}NC}\times 100\%$$

Where AG and AR refer to the regions of green and red fluorescence, respectively. Additionally, AliveNC and AliveT represent the cell viability in the control and drug-treated groups, respectively (21, 22).

### 2.7 Statistical analysis

Statistical analyses were performed using SPSS software (version 16.0), GraphPad Prism (version 8.0), and R Studio (version 4.1.2). Continuous data are presented as mean ± standard deviation. Pearson’s chi-square test was used to assess the correlation between ALG3 expression and clinical pathological features, while Cox regression models and Kaplan-Meier curves were used for survival analysis. Spearman’s rank correlation test was used to evaluate the relationship between TIIC abundance, immune checkpoint expression, and ALG3 levels. Prior to applying parametric or non-parametric tests, the normality of the data was assessed. Depending on the data distribution, either the Student’s t-test or the Wilcoxon signed-rank test was used for group comparisons. Categorical data were evaluated using the chi-square test or Fisher’s exact test. A *p*-value of <0.05 (two-tailed) was considered statistically significant. Results were derived from three independent experiments and presented as continuous quantitative measurements.

## 3 Results

### 3.1 ALG3 expression and prognosis

**Figure 1:**
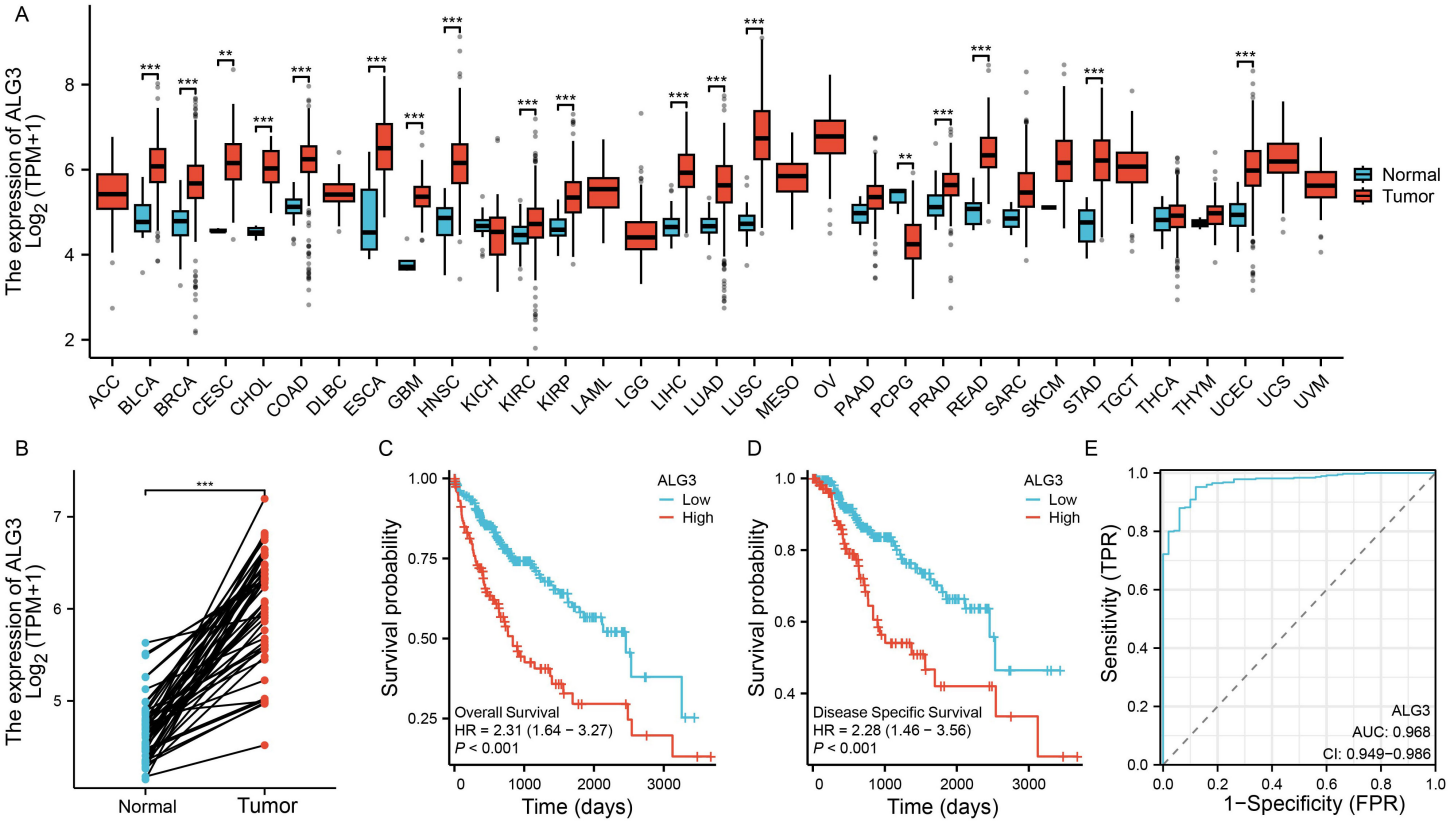
Bioinformatics analysis of ALG3 mRNA expression in hepatocellular carcinoma tissues. **(A)** Differential expression of ALG3 in different cancer tissues. **(B)** Paired expression analysis of ALG3 expression levels between tumor tissues and normal tissues in the TCGA database. **(C)** OS survival curves of patients with different ALG3 expression levels in the TCGA database. **(D)** DSS survival curves of patients with different ALG3 expression levels in the TCGA database. **(E)** ROC analysis of ALG3 as a clinical survival prognostic molecule. OS, Overall Survival, DSS, Disease-Specific Survival.**P<0.01, ***P<0.001.

To investigate the expression of ALG3 in various solid tumors, we downloaded transcriptomic data from the TCGA public database and analyzed the expression differences between cancer tissues and their corresponding normal tissues across multiple tumor types. The results indicated that ALG3 was significantly upregulated in several malignancies, including hepatocellular carcinoma (HCC), bladder cancer, breast cancer, cervical squamous cell carcinoma, cholangiocarcinoma, colon adenocarcinoma, esophageal carcinoma, head and neck squamous cell carcinoma, glioblastoma, lung adenocarcinoma, lung squamous carcinoma, prostate adenocarcinoma, rectal adenocarcinoma, gastric adenocarcinoma, and endometrial carcinoma. In contrast, ALG3 expression was downregulated in pheochromocytomas and paragangliomas (**Figure 1A**). Notably, in HCC samples, paired sample analysis was performed to compare the expression levels of ALG3 between tumor tissues and normal tissues, showing a significant upregulation of ALG3 in tumor tissues (*P* < 0.001, **Figure 1B**).

To further clarify the relationship between ALG3 expression and HCC prognosis, we selected a cohort of HCC patients with available survival information from the TCGA database. Based on the expression level of ALG3, patients were divided into high and low expression groups using the optimal cutoff value determined by the “survminer” package. Survival analysis revealed that compared to the low expression group, patients in the high expression group had significantly shorter overall survival (OS) and disease-specific survival (DSS), indicating that high ALG3 expression was significantly associated with poor prognosis (*P* < 0.001, **Figures 1C, D**). Furthermore, receiver operating characteristic (ROC) curve analysis confirmed that high ALG3 expression was an independent poor prognostic factor for OS (**Figure 1E**). In addition, we validated this finding using two external online tools, LOGpc (https://bioinfo.henu.edu.cn/index.html) and KM-Plotter (https://kmplot.com/analysis/), both of which consistently demonstrated that high ALG3 expression was associated with significantly worse overall survival in HCC patients (**Supplementary Figure S1**). These independent analyses further strengthen the prognostic value of ALG3 across different datasets.

### 3.2 Expression characteristics of ALG3 protein in HCC tissues and prognostic analysis

**Figure 2:**
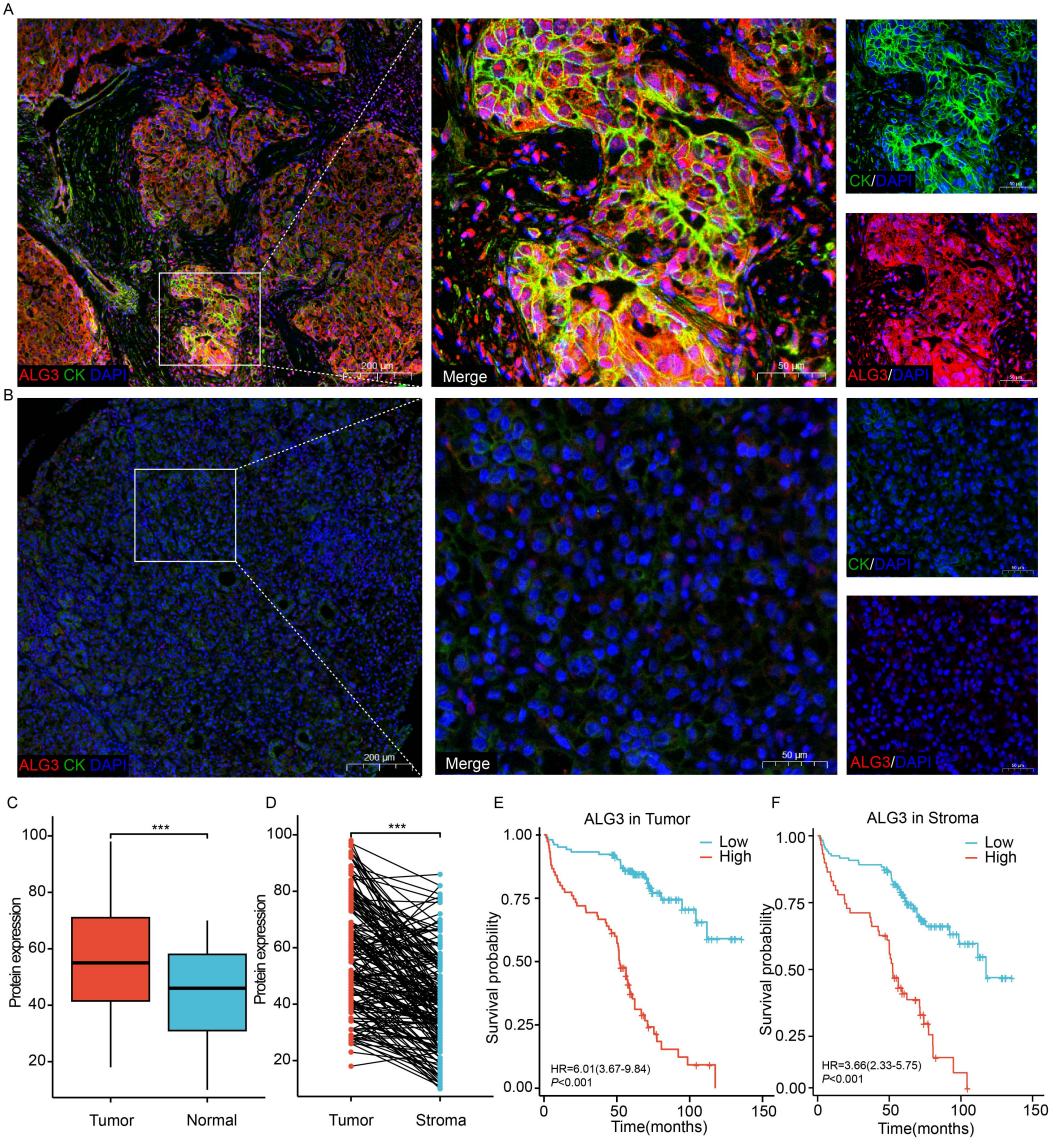
ALG3 protein expression. **(A)** ALG3 in hepatocellular carcinoma tissues. **(B)** ALG3 in non-cancerous samples. **(C)** Comparison between hepatocellular carcinoma and normal tissues. **(D)** Comparison between intra-tumoral and stromal cells. **(E)** The relationship between ALG3 protein expression in tumor cells and overall survival. **(F)** The relationship between ALG3 protein expression in stromal cells and overall survival. Red: ALG3, green: CK, blue: DAPI. DAPI: 4, 6-diamino-2-phenyl indole; CK, cytokeratin ***P<0.001.

Using the online platform OStme (https://bioinfo.henu.edu.cn/immune/immune.html), we predicted the correlation between ALG3 and immune cell infiltration at the mRNA level. The results revealed a significant positive correlation between ALG3 expression and regulatory T cells (Tregs) (R = 0.21, *P* =4.5e-05), suggesting potential immunomodulatory roles of ALG3 in HCC(**Supplementary Figure S2**).Due to the influence of post-transcriptional regulatory mechanisms, the levels of mRNA expression do not always accurately correspond to the levels of protein expression. To further evaluate the expression characteristics of ALG3 protein in HCC tissues, we employed multiplex immunohistochemistry (mIHC) for detection (**Figures 2A, B**). The results showed that the expression level of ALG3 protein in tumor cells (56.71 ± 19.12) was significantly higher than in normal epithelial cells (43.87 ± 16.50, *P* < 0.001), and the expression level of ALG3 in HCC tissues was notably higher than in benign tissues. Furthermore, ALG3 protein expression in tumor cells was significantly higher than in tumor stromal cells (40.98 ± 16.97, *P* < 0.001), suggesting that ALG3 may play an important role in the initiation and progression of HCC (**Figures 2C, D**).

To determine the optimal cutoff value for ALG3 protein expression in cancer cells, we conducted an analysis using the MaxStat package in R. The results indicated that patients were divided into high ALG3 expression group (>60, n = 75) and low expression group (≤60, n = 104). Similarly, in tumor stromal cells, patients were divided into low expression group (≤47, n = 120) and high expression group (>47, n = 59) using a cutoff value of 47.

Pearson’s χ² test revealed that high expression of ALG3 in tumor cells was significantly correlated with tumor number (*P* = 0.032), tumor size (*P* = 0.026), microvascular invasion (MVI) (*P* = 0.029), TNM clinical stage (*P* = 0.027), and CNLC stage (*P* = 0.014). Moreover, the expression of ALG3 in tumor-infiltrating immune cells (TIICs) was also significantly associated with tumor number (*P* ≤ 0.0001), CNLC stage (*P* = 0.022), and TNM stage (*P* = 0.027) (**Table 1**).

**Table 1:** Relationship between ALG3 expression and clinicopathological features.

		ALG3 in Tumor			
Characteristic	n	Low or no	High	Pearson χ2	*P*
Total	179	104 (58.1%)	75 (41.9%)		
Age				0.162	0.687
≤60	78	44 (56.4%)	34 (43.6%)		
>60	101	60 (59.4%)	41 (40.6%)		
Gender				2.306	0.129
Male	154	86 (55.8%)	68 (44.2%)		
Female	25	18 (72.0%)	7 (28.0%)		
AFP(ng/mL)				0.473	0.492
AFP ≤ 20	89	49 (55.1%)	40 (44.9%)		
AFP>20	83	50 (60.2%)	33 (39.8%)		
unknown	7				
HBV				1.299	0.254
No	42	21 (50.0%)	21 (50.0%)		
Yes	130	78 (60.0%)	52 (40.0%)		
unknown	7				
Tumor numbers				4.619	0.032^*^
Single	98	64 (65.3%)	34 (34.7%)		
Multiple	81	40 (49.4%)	41 (50.6%)		
Tumor size (cm)				4.943	0.026^*^
≤5cm	90	59 (65.6%)	31 (34.4%)		
>5cm	82	40 (48.8%)	42 (51.2%)		
unknown	7				
Tumor encapsulation				1.358	0.244
Complete	163	96 (58.9%)	67 (41.1%)		
None	14	6 (42.9%)	8 (57.1%)		
unknown	2				
CNLC stage				6.002	0.014^*^
I	138	85 (61.6%)	53 (38.4%)		
II-III	36	14 (38.9%)	22 (61.1%)		
unknown	5				
MVI				4.758	0.029^*^
No	124	77 (62.1%)	47 (37.9%)		
Yes	50	22 (44.0%)	28 (56.0%)		
unknown	5				
TNM stage				4.914	0.027^*^
I+II	147	91 (61.9%)	56 (38.1%)		
III+IV	30	12 (40.0%)	18 (60.0%)		
unknown	2				
		ALG3 in Stroma			
Characteristic	n	Low or no	High	Pearson χ2	*P*
Total	179	120 (67.0%)	59 (33.0%)		
Age				0.052	0.82
≤60	78	53 (67.9%)	25 (32.1%)		
>60	101	67 (66.3%)	34 (33.7%)		
Gender				1.056	0.304
Male	154	101 (65.6%)	53 (34.4%)		
Female	25	19 (76.0%)	6 (24.0%)		
AFP(ng/mL)				0.235	0.628
AFP ≤ 20	89	61 (68.5%)	28 (31.5%)		
AFP>20	83	54 (65.1%)	29 (34.9%)		
unknown	7				
HBV				1.35	0.245
No	42	25 (59.5%)	17 (40.5%)		
Yes	130	90 (69.2%)	40 (30.8%)		
unknown	7				
Tumor numbes				13.035	≤0.001^***^
Single	98	77 (78.6%)	21 (21.4%)		
Multiple	81	43 (53.1%)	38 (46.9%)		
Tumor size (cm)				10.155	0.001^***^
≤5cm	90	70 (77.8%)	20 (22.2%)		
>5cm	82	45(54.9%)	37 (45.1%)		
unknown	7				
Tumor encapsulation				1.9	0.168
Complete	163	111 (68.1%)	52 (31.9%)		
None	14	7 (50.0%)	7 (50.0%)		
unknown	2				
CNLC stage				5.245	0.022^*^
I	138	97 (70.3%)	41 (29.7%)		
II-III	36	18 (50.0%)	18 (50.0%)		
unknown	5				
MVI				3.189	0.074
No	124	87 (70.2%)	37 (29.8%)		
Yes	50	28 (56.0%)	22 (44.0%)		
unknown	5				
TNM stage				4.869	0.027^*^
I+II	147	104 (70.7%)	43 (29.3%)		
III+IV	30	15 (50.0%)	15 (50.0%)		
unknown	2				

^*^ *P*<0.05, ^***^ *P*<0.001.

In the univariate Cox regression analysis of 180 patients, high ALG3 expression in tumor cells, stromal cells, tumor number, tumor size, MVI, CNLC stage, and TNM stage was significantly associated with overall survival (OS). Further multivariate analysis confirmed that high expression of ALG3 in both tumor cells and stromal cells was an independent poor prognostic factor for OS (**Table 2**). Kaplan-Meier survival analysis showed that high ALG3 expression in tumor cells and TIICs was significantly associated with poorer prognosis (**Figures 2E, F**).

**Table 2:** Univariate and multivariable analyses for OS predictors in HCC patients.

Characteristic	Univariate analysis		Multivariate analysis	
	HR (95%CI)	P value	HR (95%CI)	*P* value
ALG3 in Tumor				
Low or no	Reference			
High	5.154 (3.147-8.440)	<0.001^***^	4.073 (2.411-6.882)	<0.001^***^
ALG3 in Stroma				
Low or no	Reference			
High	3.260 (2.069-5.137)	<0.001^***^	1.714 (1.059-2.776)	0.028^*^
Gender				
Male	Reference			
Female	1.196 (0.631-2.267)	0.584		
Age				
≤60	Reference			
>60	1.169 (0.745-1.835)	0.498		
AFP (ng/mL)				
≤20	Reference			
>20	1.178(0.753-1.841)	0.473		
HBV				
No	Reference			
Yes	1.152 (0.701-1.891)	0.868		
Tumor numbers				
Single	Reference			
Multiple	2.120(1.341-3.351)	0.001^***^	1.161 (0.722-1.867)	0.538
Tumor size (cm)				
≤5cm	Reference	<0.001^***^	2.646 (1.562-4.482)	0.001^***^
>5cm	3.076(1.925-4.914)			
Tumor encapsulation				
Complete	Reference			
None	1.483(0.598-3.681)	0.395		
CNLC stage				
I	Reference			
II-III	2.381 (1.466-3.867)	<0.001^***^	2.312 (1.030-5.191)	0.042^*^
MVI				
No	Reference			
Yes	1.839 (1.166-2.899)	0.009^**^	1.029 (1.618-1.715)	0.911
TNM stage				
I+II	Reference			
III+IV	1.806 (1.062-3.071)	0.029^*^	0.507(0.198-1.302)	0.158

^*^ *P*<0.05,^***^ *P*<0.001.

### 3.3 ALG3 expression correlates with the abundance of TIICs and immune checkpoints in HCC

**Figure 3:**
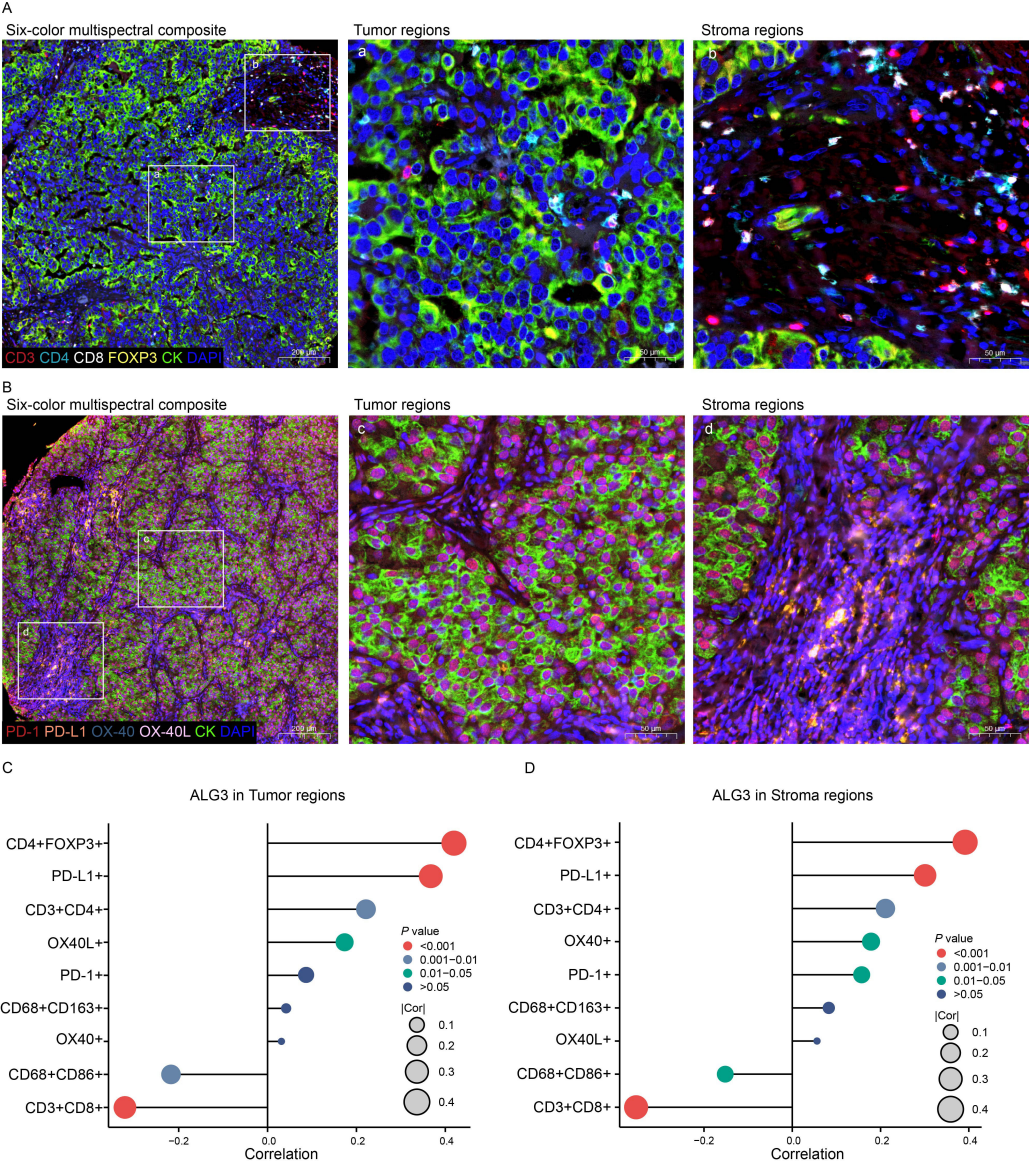
The relationship between ALG3 protein expression and tumor-infiltrating immune cells as well as immune checkpoints. **(A)** Multispectral composite of CD3, CD4, CD8, FOXP3, CK and DAPI. **(B)** Multispectral composite of PD-1, PD-L1, OX-40, OX-40L, CK and DAPI. **(C)** Correlation of ALG3 protein expression in cancer cells with immune markers. **(D)** Correlation of ALG3 protein expression in stroma cells with immune markers.

The expression levels of ALG3 in tumor cells and stromal cells were positively correlated with the abundance of CD4^+^ FOXP3^+^ regulatory T cells (Tregs), CD3^+^CD4^+^ T cells and PD-L1, whereas they were negatively correlated with the abundance of CD8^+^ T cells and CD68^+^CD86^+^ macrophages. Additionally, in stromal cells, the expression of ALG3 was also positive correlated with the abundance of OX-40 (**Figures 3A–D**).

### 3.4 ALG3 as a predictive biomarker for PD-1 resistance

**Figure 4:**
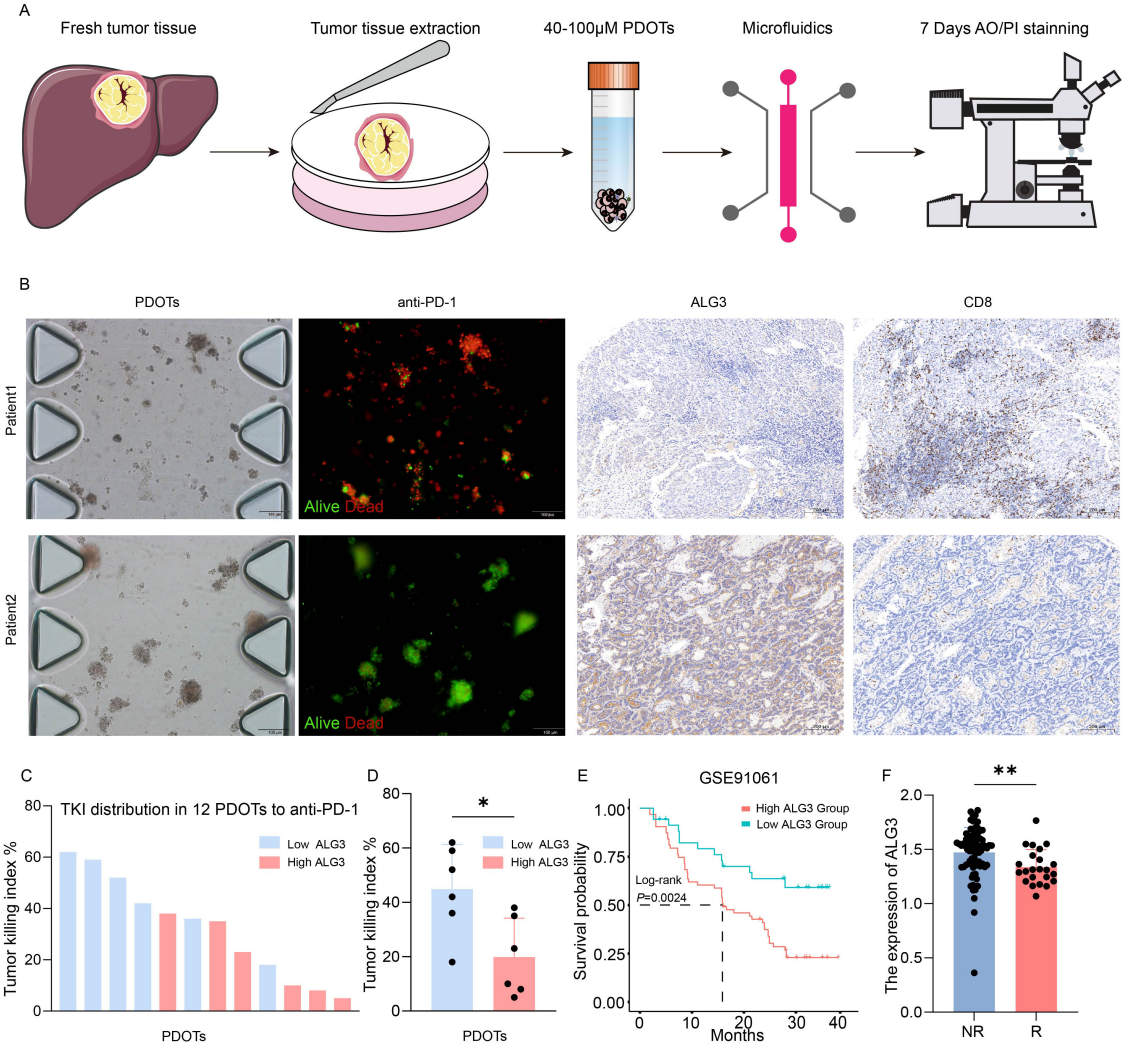
ALG3 Expression and Its Role in PD-1 Resistance in HCC. **(A)** Schematic representation and associated images of hepatocellular carcinoma **(HCC)** patient-derived organoids (PDOTs) treated with anti-PD-1, evaluated using microfluidic chips and AO/PI staining. **(B)** Microfluidic chip images of corresponding patients and the immunohistochemical expression of ALG3 and CD8. **(C)** Bar graph showing the Tumor Killing Index (TKI) of various samples from PDOTs treated with anti-PD-1. **(D)** Box plot analysis comparing the TKI response between the high and low ALG3 expression groups. Statistical significance was determined using a one-way ANOVA followed by Tukey’s *post hoc* test. **(E)** Survival curves of patients with high and low ALG3 expression in the GSE91061 dataset. **(F)** Differences in ALG3 expression between the non-responder group and the responder group. *P<0.05, **P<0.01.

In this study, 12 patients were enrolled, and patient-derived organoids (PDOTs) models were used to investigate hepatocellular carcinoma (HCC), providing a more accurate simulation of the tumor microenvironment (TME), as conventional HCC cell lines fail to adequately recapitulate the complexity of the TME. Using microfluidic chip technology, the PDOTs were treated with anti-PD-1 for one week to assess their response to PD-1 inhibition and drug sensitivity (**Figure 4A**). The clinical information of 12 patients can be found in **Supplementary Table S2**.The results revealed that tumor tissues with high ALG3 expression exhibited greater resistance to anti-PD-1 treatment, while tumor tissues with low ALG3 expression showed significantly reduced resistance (**Figure 4B**).

Additionally, in the immunohistochemical analysis of these 12 patients, ALG3 expression levels were positively correlated with the expression of CD8^+^ T cells. As shown in **Figure 4C**, the TKI (Tumor Killing Index) values for the anti-PD-1 group from the PDOTs cohort were ranked in descending order, and it was observed that patients with high ALG3 expression were predominantly clustered in the lower TKI value regions. When the 12 PDOTs samples were grouped according to ALG3 expression levels, it was evident that the low ALG3 expression group exhibited significantly higher TKI values compared to the high expression group (**Figure 4D**). Furthermore, data from the GSE91061 dataset in the GEO database revealed that patients with high ALG3 expression had poorer prognoses, and ALG3 expression was significantly higher in patients who were resistant to immunotherapy compared to those who were sensitive to immunotherapy (**Figures 4E, F**).

## 4 Discussion

Hepatocellular carcinoma (HCC) is one of the leading causes of cancer-related mortality worldwide, with its high heterogeneity and treatment resistance significantly limiting clinical efficacy (23). Despite progress in targeted therapy and immunotherapy in recent years, the complexity of the tumor and individual differences still lead to limited effectiveness (23, 24). This study, by integrating bioinformatic analyses, multiplex immunohistochemistry, and patient-derived organoid models, for the first time reveals the critical role of ALG3, a key enzyme involved in endoplasmic reticulum glycosylation—in reshaping the HCC immune microenvironment and mediating resistance to PD-1 blockade. These findings provide important insights into the immune evasion mechanisms in HCC and offer new avenues for therapeutic strategies.

Analysis based on TCGA data shows that ALG3 is significantly upregulated in HCC tissues and is closely associated with shorter overall survival (OS) and disease-free survival (DSS), highlighting its potential as a prognostic biomarker. Further clinical correlation analysis reveals that high ALG3 expression is significantly associated with tumor size, tumor stage, microvascular invasion (MVI), and clinical staging, all of which are key predictors of HCC progression and prognosis. ALG3 may regulate these tumor biological features and directly contribute to tumor invasiveness and metastasis, consistent with findings from Zhao et al., indicating that ALG3 is an independent adverse prognostic factor (25). Therefore, ALG3 is not only an independent prognostic factor for HCC but also a potential target for targeted therapy.

Treg cells inhibit the activation and cytotoxic functions of CD8^+^ T cells by secreting immune-suppressive cytokines such as IL-10 and TGF-β (26, 27). This study confirms that high ALG3 expression significantly increases Treg cell infiltration, suggesting that ALG3 may play a role in HCC immune evasion (28, 29). The high levels of Treg cells in the tumor microenvironment have been widely implicated in immune evasion and poor prognosis. Therefore, ALG3, by enhancing Treg cell infiltration, may suppress anti-tumor immune responses, promoting HCC progression.

In addition, this study found a negative correlation between ALG3 and CD68^+^CD86^+^ macrophages. M1 macrophages are classical immune-activated macrophages that provide co-stimulatory signals through CD86, which binds to the CD28 receptor on T cells, leading to T cell activation and promoting anti-tumor immune responses (30). M1 macrophages exhibit a strong pro-inflammatory profile, secreting cytokines such as TNF-α, IL-1β, and IL-6, thereby activating additional immune cells, especially CD8^+^ T cells, and enhancing tumor immune surveillance. However, high expression of ALG3 may suppress the function of CD68^+^CD86^+^ macrophages or reduce their recruitment in the tumor microenvironment, thereby weakening the anti-tumor immune response (31). ALG3 may influence macrophage polarization, potentially promoting the recruitment of M2 macrophages, which are involved in immune suppression, aiding tumor immune escape, and facilitating tumor growth and metastasis (32). Therefore, ALG3 plays a critical role not only in the initiation and progression of hepatocellular carcinoma but also in modulating the immune microenvironment. By affecting the function of M1 macrophages, ALG3 may contribute to immune escape and immune suppression within the tumor, providing a potential therapeutic target for overcoming immune evasion.

Moreover, this study also discovered a positive correlation between ALG3 and the immune activation molecule OX40. ALG3 may regulate OX40 through glycosylation modifications, thereby limiting T cell function, particularly in the highly immune-tolerant tumor environment of HCC. According to the study by Xie et al., the expression of OX40 in HCC tumor tissues is significantly higher than in adjacent normal tissues, and high OX40 expression is closely associated with elevated serum AFP, vascular invasion, and poor prognosis. Moreover, tumors with high OX40 expression exhibit characteristics of an immunosuppressive microenvironment, including the enrichment of exhausted CD8^+^ T cells and activation of the AKT/mTOR pathway mutations (33). This mechanism not only enhances the tumor’s immune evasion properties but also provides a theoretical basis for future strategies targeting ALG3 to reverse T cell exhaustion and improve the effectiveness of immunotherapy.

Although PD-1 inhibitors have made some progress in HCC treatment, resistance remains a significant issue (34, 35). This study finds that high ALG3 expression correlates positively with PD-L1, suggesting that ALG3 may influence the stability or membrane localization of PD-L1 through glycosylation modifications, thereby indirectly diminishing the inhibitory effect of the PD-1/PD-L1 pathway and promoting immune evasion. This mechanism is closely related to the variability in the effectiveness of current PD-1 inhibitor treatments and may provide new insights into tumor resistance to immune checkpoint inhibitors.

Similar studies have shown that ALG3 is highly expressed in various cancer types and is closely associated with changes in the immune microenvironment. In a study on triple-negative breast cancer (TNBC), high ALG3 expression was found to correlate with poor clinical prognosis and immune evasion. ALG3 suppressed the secretion of chemotactic factors, reducing CD8^+^ T cell infiltration, and affected the efficacy of immunotherapy (9, 36). Moreover, inhibition of ALG3 enhanced the sensitivity of TNBC cells to chemotherapy with 5-fluorouracil, further emphasizing the potential of ALG3 as a biomarker for immune therapy resistance.

In the PDOTs model, organoids with high ALG3 expression exhibited significantly reduced sensitivity to anti-PD-1 treatment, accompanied by decreased CD8^+^ T cell infiltration. These results suggest that ALG3 may serve as a biomarker for predicting resistance to PD-1 inhibitors and provide a theoretical basis for personalized treatment strategies combining targeting of ALG3 with immune checkpoint inhibitors (ICIs).

From a clinical translation perspective, ALG3 is not only an independent prognostic factor for HCC but may also be an important target for future immunotherapies. Based on the findings of this study, high ALG3 expression may provide a new biomarker for tumors resistant to anti-PD-1 therapy and offer a theoretical foundation for the development of personalized immunotherapy strategies. Particularly, targeting ALG3 in combination with immune checkpoint inhibitors may become a novel therapeutic approach.

The strength of this study lies in its systematic integration of bioinformatics, multiplex immunohistochemistry, and preclinical models, comprehensively validating the biological function of ALG3 in the HCC immune microenvironment. Future studies can further explore the specific molecular mechanisms by which ALG3 regulates the immune microenvironment, such as its glycosylation modifications of target proteins. Additionally, the sample size of the PDOTs model was relatively small (n = 12). To improve the generalizability of the conclusions, future studies should expand the sample size and include patients at different clinical stages for validation. Moreover, although we comprehensively analyzed the association between ALG3 and various immune cell types—including Tregs, CD4^+^ T cells, CD8^+^ T cells, and different macrophage subsets—using mIHC, the validation in the PDOTs model focused solely on CD8^+^ T cells. This decision was primarily influenced by practical limitations such as limited tissue availability, restricted antibody multiplexing capacity, and the technical compatibility of the microfluidic chip platform. CD8^+^ T cells were prioritized due to their pivotal role in tumor immunity and their direct relevance to PD-1 blockade response. In future studies, we plan to incorporate more sophisticated multiplex immunostaining techniques or spatial transcriptomic analyses into the PDOTs model, along with dynamic tracking of immune responses before and after treatment within the same sample, to achieve a more comprehensive understanding of the immunoregulatory role of ALG3. Furthermore, whether ALG3 regulates other immune checkpoints (e.g., LAG-3 or TIM-3) to participate in resistance remains to be explored.

## Data Availability

The raw data supporting the conclusions of this article will be made available by the authors, without undue reservation.

## Supplementary material

Supplementary Figure 1 Prognostic analysis of ALG3 mRNA expression in hepatocellular carcinoma (HCC) using two independent online databases. **(A)** Kaplan-Meier survival curves generated using the LOGpc platform (https://bioinfo.henu.edu.cn/index.html). **(B)** Kaplan-Meier survival curves generated using the KM-Plotter platform (https://kmplot.com/analysis/).

Supplementary Figure 2 Correlation between ALG3 mRNA expression and the abundance of tumor-infiltrating immune cells (TIICs) in hepatocellular carcinoma tissues. Panels **(A–V)** show the predicted associations between ALG3 expression and various immune cell populations based on the OStme platform (https://bioinfo.henu.edu.cn/Immune/Immune.html), using TCGA-LIHC dataset.
