# Supplementary figures and images for: ALG3 as a prognostic biomarker and mediator of PD-1 blockade resistance in hepatocellular carcinoma
Source: Front Immunol. 2025 May 22;16:1589153. doi: 10.3389/fimmu.2025.1589153 (PMC12137335; doi:10.3389/fimmu.2025.1589153)

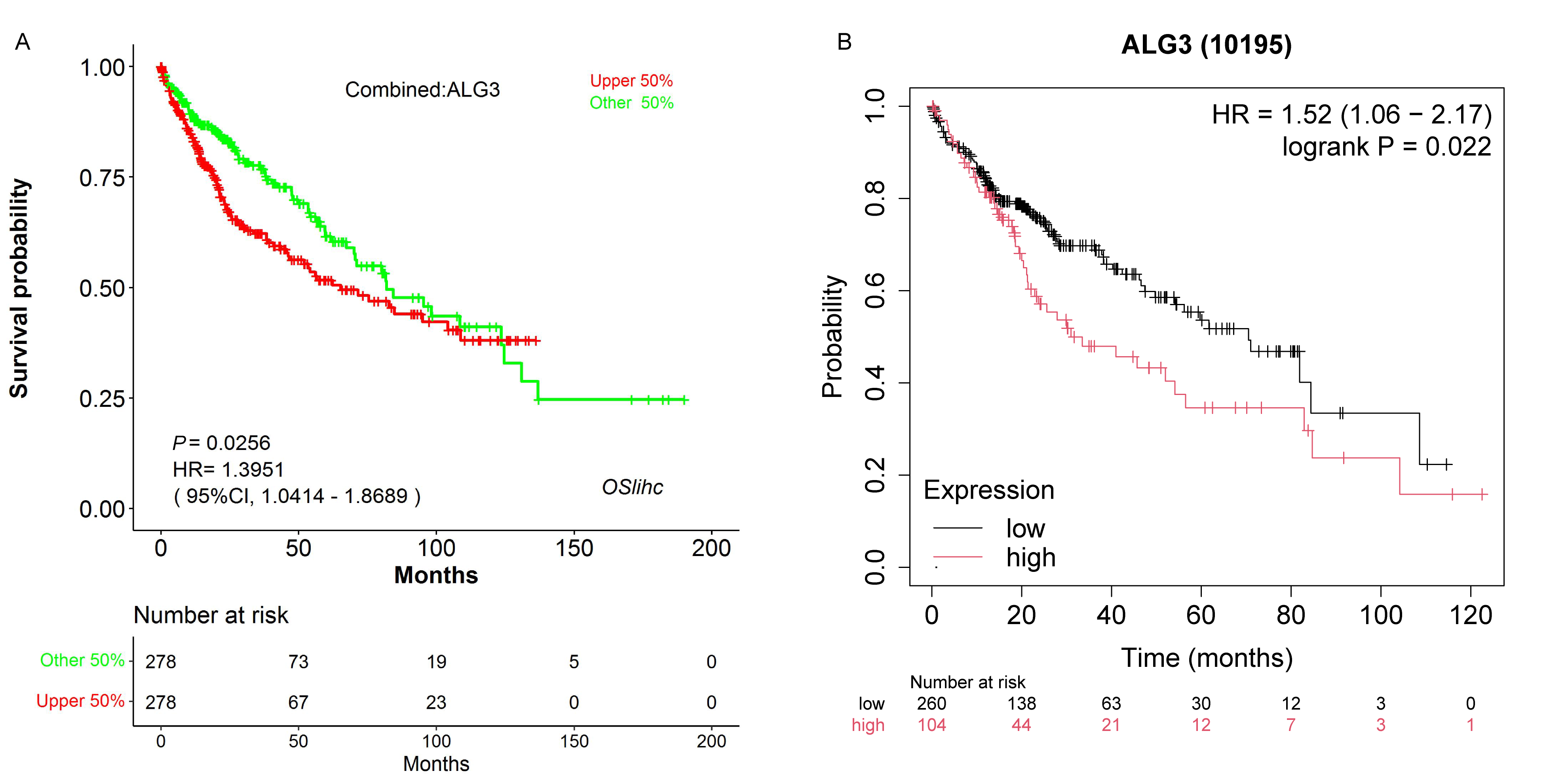

Supplement: Supplementary Figure 1 — Prognostic analysis of ALG3 mRNA expression in hepatocellular carcinoma (HCC) using two independent online databases. (A) Kaplan-Meier survival curves generated using the LOGpc platform (https://bioinfo.henu.edu.cn/index.html). (B) Kaplan-Meier survival curves generated using the KM-Plotter platform (https://kmplot.com/analysis/). [file Image1.tif]

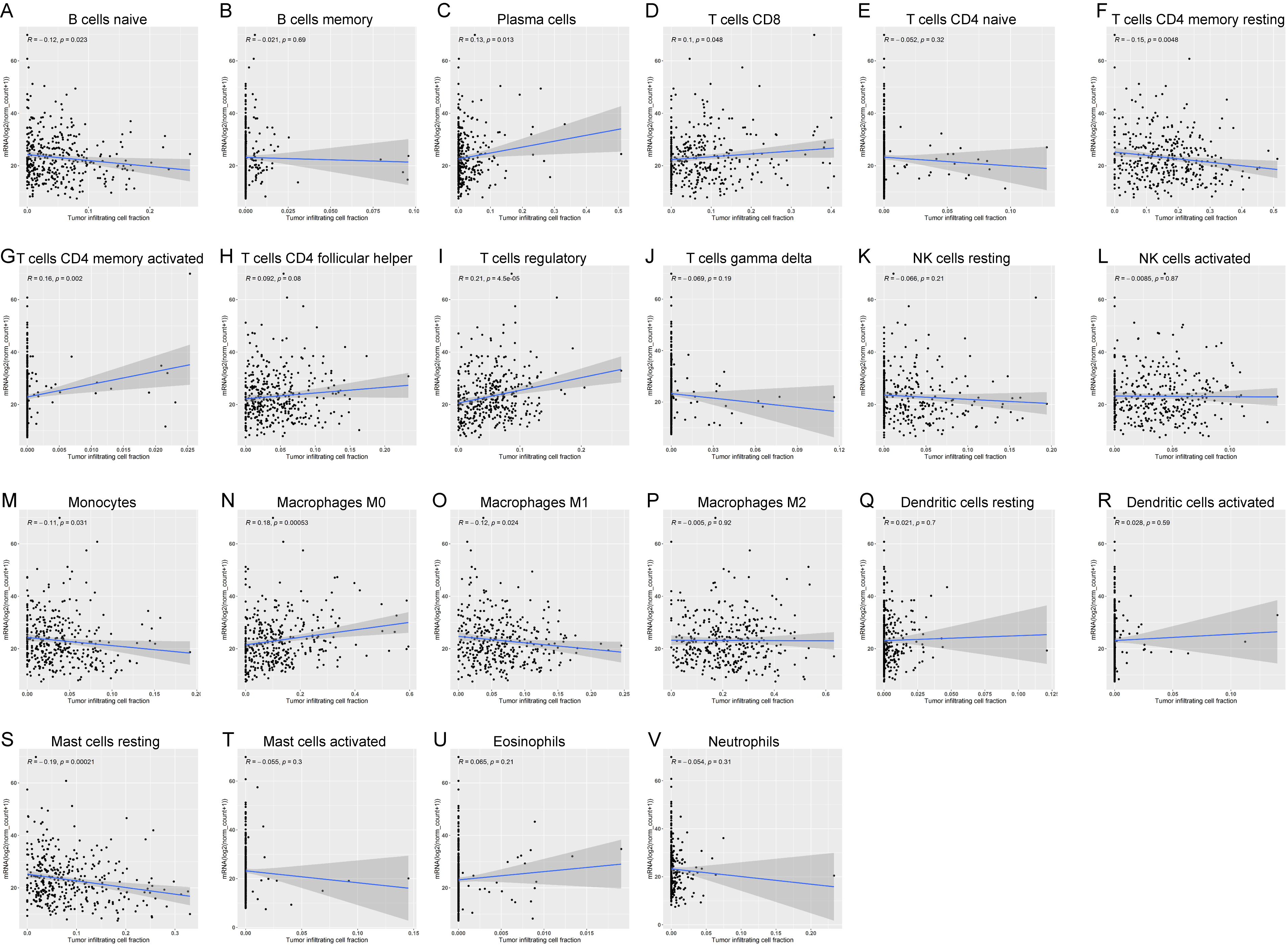

Supplement: Supplementary Figure 2 — Correlation between ALG3 mRNA expression and the abundance of tumor-infiltrating immune cells (TIICs) in hepatocellular carcinoma tissues. Panels (A–V) show the predicted associations between ALG3 expression and various immune cell populations based on the OStme platform (https://bioinfo.henu.edu.cn/Immune/Immune.html), using TCGA-LIHC dataset. [file Image2.tif]
