# Supplementary material for: ALG3 as a prognostic biomarker and mediator of PD-1 blockade resistance in hepatocellular carcinoma
Source: Front Immunol. 2025 May 22;16:1589153. doi: 10.3389/fimmu.2025.1589153 (PMC12137335; doi:10.3389/fimmu.2025.1589153)
Supplement: Supplementary file 4 [file Table2.docx]

| **Table S2. Baseline Clinical Characteristics of 12 Patients in the Microfluidic PDOTs Model.** | | | | | |
| --- | --- | --- | --- | --- | --- |
| **Variable** |  | **Number of Patients** | | | |
|  |  | **ALG3**  **Low(N=6)** | **ALG3**  **High(N=6)** | **Total(N=12)** | **P Value** |
| Age |  |  |  |  | 0.567 |
|  | ≤60 | 4（66.7%） | 2(33.3%) | 6(50.0%) |  |
|  | ＞60 | 2(33.3%) | 4（66.7%） | 6(50.0%) |  |
| Gender |  |  |  |  | 1 |
|  | Male | 3(50.0%) | 3(50.0%) | 6(50.0%) |  |
|  | Female | 3(50.0%) | 3(50.0%) | 6(50.0%) |  |
| Liver cirrhosis |  |  |  |  | 1 |
|  | No | 3（42.9%） | 4（57.1%） | 7(58.3%) |  |
|  | Yes | 3（60.0%） | 2（40.0%） | 5(41.7%) |  |
| HBsAg |  |  |  |  | 0.080 |
|  | Negative | 5（83.3%） | 1（16.7%） | 6(50.0%) |  |
|  | Positive | 1（16.7%） | 5（83.3%） | 6(50.0%) |  |
| HCV |  |  |  |  | 0.182 |
|  | Negative | 6(66.7%) | 3(33.3%) | 9(75.0%) |  |
|  | Positive | 0(0.0e+0%) | 3(100.0%) | 3(25.0%) |  |
| Serum AFP, ng/mL |  |  |  |  | 0.061 |
|  | <20 | 4(100.0%) | 0(0.0e+0%) | 4(33.3%) |  |
|  | ≥20 | 2(25.0%) | 6(75.0%) | 8(66.7%) |  |
| Tumor size |  |  |  |  | 0.061 |
|  | <5 (CM) | 4(100.0%) | 0(0.0e+0%) | 4(33.3%) |  |
|  | ≥5 (CM) | 2(25.0%) | 6(75.0%) | 8(66.7%) |  |
| Tumor number |  |  |  |  | 1 |
|  | Single | 3(60.0%) | 2(40.0%) | 5(41.7%) |  |
|  | Multiple | 3(42.9%) | 4(57.1%) | 7(58.3%) |  |
| Encapsulation invasion |  |  |  |  | 0.242 |
|  | No | 1(20.0%) | 4(80.0%) | 5(41.7%) |  |
|  | Yes | 5(71.4%) | 2(28.6%) | 7(58.3%) |  |
| Pathologic_stage |  |  |  |  | 0.545 |
|  | I-II | 3(75.0%) | 1(25.0%) | 4(33.3%) |  |
|  | III-IV | 3(37.5%) | 5(62.5%) | 8(66.7%) |  |
| TNM stage |  |  |  |  | 0.545 |
|  | I-Ⅲ | 3(37.5%) | 5(62.5%） | 8(66.7%) |  |
|  | Ⅳ | 3（74.0%） | 1（25.0%） | 4(33.3%) |  |
